# 2-[(Meth­oxy­carbonothio­yl)sulfan­yl]acetic acid

**DOI:** 10.1107/S1600536811003941

**Published:** 2011-02-05

**Authors:** Shude Xiao, Paul A. Charpentier

**Affiliations:** a Dept. of Chemical and Biochemical Engineering, Faculty of Engineering, The University of Western Ontario, London, Ontario, Canada N6A 5B9

## Abstract

The title compound, C_4_H_6_O_3_S_2_, features a characteristic xanthate group; the C=S double bond is shorter than the C—S single bond, and the methyl group is coplanar with the xanthate group. In the crystal pairs of mol­ecules form dimers through inter­molecular O—H⋯O hydrogen bonding.

## Related literature

For a related structure, see: Xiao & Charpentier (2010 ▶). For the design and applications of the title compound, see: Moad *et al.* (2005 ▶, 2008 ▶); Stenzel *et al.* (2003 ▶); Coote & Radom (2004 ▶); Coote *et al.* (2006 ▶).
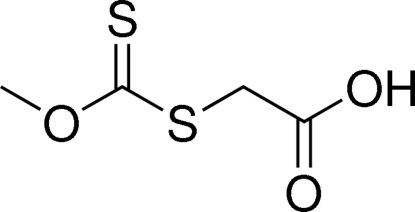


## Experimental

### 

#### Crystal data

C_4_H_6_O_3_S_2_
*M* *_r_* = 166.21
Monoclinic, 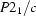

*a* = 7.1009 (3) Å
*b* = 10.6485 (5) Å
*c* = 9.2022 (4) Å
β = 93.370 (1)°
*V* = 694.61 (5) Å^3^
*Z* = 4
Mo *K*α radiation
μ = 0.70 mm^−1^
*T* = 150 K
0.10 × 0.07 × 0.06 mm

#### Data collection

Bruker APEXII CCD diffractometer
Absorption correction: multi-scan (*SADABS*; Sheldrick, 1996 ▶) *T*_min_ = 0.931, *T*_max_ = 0.963
33976 measured reflections
1723 independent reflections
1517 reflections with *I* > 2σ(*I*)
*R*_int_ = 0.038

#### Refinement

*R*[*F* ^2^ > 2σ(*F* ^2^)] = 0.021
*wR*(*F* ^2^) = 0.056
*S* = 1.05
1723 reflections
84 parameters
H-atom parameters constrained
Δρ_max_ = 0.29 e Å^−3^
Δρ_min_ = −0.20 e Å^−3^

### 

Data collection: *APEX2* (Bruker, 2009 ▶); cell refinement: *SAINT* (Bruker, 2009 ▶); data reduction: *SAINT*; program(s) used to solve structure: *SHELXS97* (Sheldrick, 2008 ▶); program(s) used to refine structure: *SHELXL97* (Sheldrick, 2008 ▶); molecular graphics: *SHELXTL* (Sheldrick, 2008 ▶); software used to prepare material for publication: *SHELXTL*.

## Supplementary Material

Crystal structure: contains datablocks global, I. DOI: 10.1107/S1600536811003941/ng5085sup1.cif

Structure factors: contains datablocks I. DOI: 10.1107/S1600536811003941/ng5085Isup2.hkl

Additional supplementary materials:  crystallographic information; 3D view; checkCIF report

## Figures and Tables

**Table 1:** Hydrogen-bond geometry (Å, °)

*D*—H⋯*A*	*D*—H	H⋯*A*	*D*⋯*A*	*D*—H⋯*A*
O2—H2⋯O3^i^	0.84	1.82	2.6540 (12)	175

Symmetry code: (i) 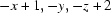.

**Fig. 1.**
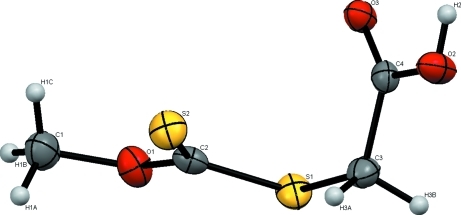
View of the title compound (50% probability displacement ellipsoids).

**Fig. 2.**
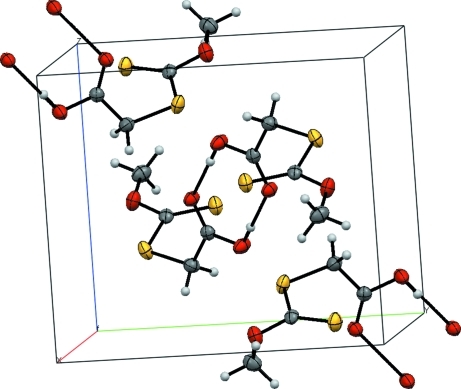
Packing diagram of the structure with H-bonds.

## supplementary crystallographic information

### Comment

Carbonothioylthio (S=C—S) compounds are used as chain transfer agents (CTA) in addition-fragmentation chain-transfer (RAFT) polymerization. In the addition-fragmentation equilibria, addition of the propagating radicals to the S=C group followed by fragmentation of the intermediate radical at the C—S bond generates a new radical and a polymeric carbonothioylthio compound (Moad *et al.*, 2005, 2008). *O*-alkyl xanthates show low reactivity in RAFT equilibria due to the conjugation of the *O* lone pair electrons and the C=S bond which is favorable to the zwitterionic canonical forms of xanthates (Moad *et al.*, 2005; Coote *et al.*, 2006). However, xanthates can promote fragmentation of unstable radicals, such as vinyl acetate radicals that undergo fast addition and slow fragmentation (Coote *et al.*, 2006). Though studies have been done on RAFT polymerization of vinyl acetate with methyl 2-(methoxycarbonothioylthio)acetate (Stenzel *et al.*, 2003; Coote & Radom, 2004), 2-(methoxycarbonothioylthio)acetic acid has not been used in RAFT polymerization. Therefore, efforts were made to use 2-(methoxycarbonothioylthio)acetic acid as the CTA in RAFT polymerization, and poly(vinyl acetate)s containing carboxylic acid end groups were successfully prepared. A similar compound, 2-(isopropoxycarbonothioylthio)acetic acid, has been reported for the same application (Xiao & Charpentier, 2010).

### Experimental

Potassium hydroxide 5.6 g (50 mmol) was dissolved in methanol 30 ml at room temperature. The solution was cooled with an ice bath when carbon disulfide 20 ml was charged into the flask dropwise. After 1 day reaction at room temperature, a solution of 2-bromoacetic acid 6.9 g (50 mmol) / methanol 20 ml was added into the flask dropwise in an ice bath. The precipitates were removed by filtration after 2 days reaction at room temperature, and the solvent was evaporated with a rotary evaporator. The crude product was run through a silica gel column with a mixture of ethyl ether / hexanes (5:1). Colorless crystals were obtained from crystalization in hexanes/ cyclohexane (4:1). m.p.: 112.6 °C (DSC). MS: 165.9764.

### Refinement

The structure was solved and refined using the Bruker *SHELXTL* Software Package, using the space group P 1 21/c 1, with *Z* = 4 for the formula unit, C_4_H_6_O_3_S_2_. All of the non-hydrogen atoms were refined with anisotropic thermal parameters. The hydrogen atom positions were calculated geometrically and were included as riding on their respective carbon/oxygen atoms. The final anisotropic full-matrix least-squares refinement on F^2^ with 84 variables converged at R1 = 2.13%, for the observed data and wR2 = 5.55% for all data. The goodness-of-fit was 1.047. The largest peak in the final difference electron density synthesis was 0.288 e^-^/Å^3^ and the largest hole was -0.195 e^-^/Å^3^ with an RMS deviation of 0.040 e^-^/Å^3^. On the basis of the final model, the calculated density was 1.589 g/cm^3^ and F(000), 344 e^-^.

### Crystal data

**Table:**

C_4_H_6_O_3_S_2_	*F*(000) = 344
*M**_r_* = 166.21	*D*_x_ = 1.589 Mg m^−^^3^
Monoclinic, *P*2_1_/*c*	Mo *K*α radiation, λ = 0.71073 Å
Hall symbol: -P 2ybc	Cell parameters from 9941 reflections
*a* = 7.1009 (3) Å	θ = 2.9–30.2°
*b* = 10.6485 (5) Å	µ = 0.70 mm^−^^1^
*c* = 9.2022 (4) Å	*T* = 150 K
β = 93.370 (1)°	Block, colourless
*V* = 694.61 (5) Å^3^	0.10 × 0.07 × 0.06 mm
*Z* = 4	

### Data collection

**Table:**

Bruker APEXII CCD diffractometer	1723 independent reflections
Radiation source: fine-focus sealed tube	1517 reflections with *I* > 2σ(*I*)
graphite	*R*_int_ = 0.038
φ and ω scans	θ_max_ = 28.3°, θ_min_ = 2.9°
Absorption correction: multi-scan (*SADABS*; Sheldrick, 1996)	*h* = −9→9
*T*_min_ = 0.931, *T*_max_ = 0.963	*k* = −14→13
33976 measured reflections	*l* = −12→12

### Refinement

**Table:**

Refinement on *F*^2^	Primary atom site location: structure-invariant direct methods
Least-squares matrix: full	Secondary atom site location: difference Fourier map
*R*[*F*^2^ > 2σ(*F*^2^)] = 0.021	Hydrogen site location: inferred from neighbouring sites
*wR*(*F*^2^) = 0.056	H-atom parameters constrained
*S* = 1.05	*w* = 1/[σ^2^(*F*_o_^2^) + (0.0234*P*)^2^ + 0.2541*P*] where *P* = (*F*_o_^2^ + 2*F*_c_^2^)/3
1723 reflections	(Δ/σ)_max_ = 0.001
84 parameters	Δρ_max_ = 0.29 e Å^−^^3^
0 restraints	Δρ_min_ = −0.20 e Å^−^^3^

### Special details

**Table:**

Geometry. All e.s.d.'s (except the e.s.d. in the dihedral angle between two l.s. planes) are estimated using the full covariance matrix. The cell e.s.d.'s are taken into account individually in the estimation of e.s.d.'s in distances, angles and torsion angles; correlations between e.s.d.'s in cell parameters are only used when they are defined by crystal symmetry. An approximate (isotropic) treatment of cell e.s.d.'s is used for estimating e.s.d.'s involving l.s. planes.
Refinement. Refinement of *F*^2^ against ALL reflections. The weighted *R*-factor *wR* and goodness of fit *S* are based on *F*^2^, conventional *R*-factors *R* are based on *F*, with *F* set to zero for negative *F*^2^. The threshold expression of *F*^2^ > σ(*F*^2^) is used only for calculating *R*-factors(gt) *etc*. and is not relevant to the choice of reflections for refinement. *R*-factors based on *F*^2^ are statistically about twice as large as those based on *F*, and *R*- factors based on ALL data will be even larger.

### Fractional atomic coordinates and isotropic or equivalent isotropic displacement parameters (Å^2^)

**Table:**

	*x*	*y*	*z*	*U*_iso_*/*U*_eq_	
S1	0.15918 (5)	0.31498 (3)	0.78725 (4)	0.02980 (9)	
S2	−0.11784 (5)	0.12843 (3)	0.90015 (4)	0.03122 (9)	
O1	−0.10815 (13)	0.37609 (8)	0.93394 (10)	0.0311 (2)	
O2	0.41851 (14)	−0.01297 (9)	0.81022 (10)	0.0319 (2)	
H2	0.4754	−0.0511	0.8797	0.048*	
O3	0.38409 (12)	0.13435 (8)	0.98001 (9)	0.02570 (19)	
C1	−0.2814 (2)	0.36782 (14)	1.00825 (15)	0.0354 (3)	
H1A	−0.3839	0.3404	0.9395	0.053*	
H1B	−0.3120	0.4504	1.0476	0.053*	
H1C	−0.2659	0.3070	1.0880	0.053*	
C2	−0.03785 (16)	0.27021 (11)	0.88181 (13)	0.0233 (2)	
C3	0.25018 (18)	0.16615 (12)	0.73538 (13)	0.0279 (3)	
H3A	0.1438	0.1134	0.6969	0.034*	
H3B	0.3354	0.1794	0.6554	0.034*	
C4	0.35645 (16)	0.09552 (11)	0.85644 (13)	0.0224 (2)	

### Atomic displacement parameters (Å^2^)

**Table:**

	*U*^11^	*U*^22^	*U*^33^	*U*^12^	*U*^13^	*U*^23^
S1	0.03072 (17)	0.02100 (15)	0.03800 (18)	0.00050 (12)	0.00457 (13)	0.00871 (12)
S2	0.03306 (17)	0.02151 (16)	0.03927 (19)	−0.00432 (12)	0.00377 (13)	0.00243 (12)
O1	0.0293 (5)	0.0230 (4)	0.0407 (5)	0.0011 (3)	0.0005 (4)	−0.0062 (4)
O2	0.0404 (5)	0.0291 (5)	0.0252 (4)	0.0120 (4)	−0.0050 (4)	−0.0049 (4)
O3	0.0264 (4)	0.0255 (4)	0.0248 (4)	0.0046 (3)	−0.0011 (3)	−0.0034 (3)
C1	0.0337 (7)	0.0385 (7)	0.0339 (7)	0.0061 (6)	0.0026 (6)	−0.0058 (6)
C2	0.0245 (6)	0.0223 (6)	0.0224 (5)	0.0007 (4)	−0.0060 (4)	0.0009 (4)
C3	0.0317 (6)	0.0281 (6)	0.0242 (6)	0.0041 (5)	0.0040 (5)	0.0041 (5)
C4	0.0195 (5)	0.0226 (5)	0.0254 (6)	−0.0001 (4)	0.0040 (4)	0.0005 (4)

### Geometric parameters (Å, °)

**Table:**

S1—C2	1.7564 (13)	O3—C4	1.2150 (14)
S1—C3	1.7870 (13)	C1—H1A	0.9800
S2—C2	1.6253 (12)	C1—H1B	0.9800
O1—C2	1.3336 (15)	C1—H1C	0.9800
O1—C1	1.4451 (17)	C3—C4	1.5091 (16)
O2—C4	1.3159 (14)	C3—H3A	0.9900
O2—H2	0.8400	C3—H3B	0.9900
			
C2—S1—C3	101.69 (6)	S2—C2—S1	126.65 (7)
C2—O1—C1	117.76 (10)	C4—C3—S1	114.70 (9)
C4—O2—H2	109.5	C4—C3—H3A	108.6
O1—C1—H1A	109.5	S1—C3—H3A	108.6
O1—C1—H1B	109.5	C4—C3—H3B	108.6
H1A—C1—H1B	109.5	S1—C3—H3B	108.6
O1—C1—H1C	109.5	H3A—C3—H3B	107.6
H1A—C1—H1C	109.5	O3—C4—O2	124.23 (11)
H1B—C1—H1C	109.5	O3—C4—C3	124.58 (11)
O1—C2—S2	127.40 (10)	O2—C4—C3	111.18 (10)
O1—C2—S1	105.94 (8)		

### Hydrogen-bond geometry (Å, °)

**Table:**

*D*—H···*A*	*D*—H	H···*A*	*D*···*A*	*D*—H···*A*
O2—H2···O3^i^	0.84	1.82	2.6540 (12)	175

Symmetry codes: (i) −*x*+1, −*y*, −*z*+2.
